# Effect of pre-treatment and drying methods on the content of minerals, B-group vitamins and tocopherols in kale (*Brassica oleracea* L. var. *acephala*) leaves

**DOI:** 10.1007/s13197-021-05012-9

**Published:** 2021-02-10

**Authors:** Anna Korus

**Affiliations:** Department of Plant Product Technology and Nutrition Hygiene, Faculty of Food Technology, University of Agriculture in Krakow, Balicka 122 Street, 30-149 Krakow, Poland

**Keywords:** Kale, Blanching, Drying, Minerals, B-vitamins, Tocopherols

## Abstract

Dried vegetables are widely used in food production. Kale leaves, due to their high health-promoting properties, can be a valuable raw material for drying. The aim of the study was to evaluate the effect of blanching, drying methods (air-drying, freeze-drying), the time and temperature of storage on the content of ash, minerals, vitamins B_1_, B_2_, and tocopherols in dried kale products. The dried products were evaluated directly after processing and after 12 months of storage at ambient temperature, 18–20 °C and cold temperature, 8–10 °C. In 100 g dry matter from fresh raw material the dominant minerals were potassium (2613 mg), calcium (1346 mg), phosphorus (432 mg), magnesium (129 mg). Kale leaves had 0.828 mg vitamin B_1_, 1.533 mg vitamin B_2_ and 16.55 mg total tocopherols per 100 g of dry matter. Blanching, applied prior to drying, significantly reduced the levels of minerals (3–38%) and vitamins (8–45%), except for calcium, zinc and manganese. This pre-treatment had, however, a beneficial effect, especially on vitamin retention during the storage of dried products. After 12 month storage, the losses of vitamin B_1_, B_2_ and total tocopherols in dried, previously blanched, raw materials ranged from 3 to 10%, 1 to 4%, and 1 to 16%, respectively, depending on the type of sample. In the dried products obtained from unblanched raw material, the losses were larger and amounted to 10–17%, 8–16% and 4–17%, respectively. Throughout drying and storage, the minerals examined were fairly stable. Dried kale is generally a good source of minerals and vitamins. However, blanching before drying is recommended. In addition, freeze-drying and storage in cold temperature allows for higher vitamin preservation in dried kale.

## Introduction

Diet high in vegetables is widely recommended for its health-promoting properties. Vegetables are consumed raw or numerous processed products. Intake of vegetables has been associated with a reduced risk of chronic diseases and body weight management (Pem and Jeewon 2015). Most of the vegetables include high content of non-nutritive, nutritive, and bioactive compounds such as flavonoids, phenolic acids, anthocyanins, and as well as nutritive compounds such as sugars, essential oils, carotenoids, vitamins, and minerals (Arin and Arabaci 2019; Pennington and Fisher 2009; Zia-Ul-Haq et al. 2013). Studies indicate that human nutrition should be based on fruit and vegetables. These provide many valuable substances and significantly reduce the incidence of cardiovascular disease and diabetes, along with the associated mortality (Aune et al. 2017; Ülger et al. 2018). Therefore, we should eat fruit and vegetables as often as possible to improve overall health and reduce the risk of cardiovascular diseases and certain types of cancer. FAO/WHO (2015) suggests an intake of over 400 g of fruit and vegetables per day, which translates to about 5 portions per day. In addition, FAO/WHO have been leading the global initiative ‘Promotion of Fruit and Vegetables for Health’ (PROFAV) to raise awareness and to boost fruit and vegetable production, supply and consumption, in order to improve people's health (FAO/WHO 2015). It should also be emphasised that nutritionists recommend eating as much green vegetables as possible as these are a source of many valuable components, for example, lutein, vitamin K_1_ (phylloquinone), folate, α-tocopherol, β-carotene, and the flavonoid kaempferol.

Among green vegetables, kale (*Brassica oleracea* L. var. *acephala*) deserves special attention. It is rich in bioactive phytochemicals, such as phenolic compounds, glucosinolates and vitamins (Korus et al. 2014). Kale is an excellent source of vitamin K and a good source of omega-3 fatty acids (in the form of alpha-linolenic acid) (Capurso et al. 2018). This species is also characterised by a high content of minerals (Armesto et al. 2019). Thavarajah et al. (2016) showed that a single 100 g serving of fresh kale can provide a significant percentage of the recommended daily intake of mineral micronutrients (188–873 mg K, 35–300 mg Ca and 20–100 mg Mg) identified by the United States Department of Agriculture (USDA 2015).

Kale is a seasonal vegetable and is susceptible to deterioration after harvesting due to its high water content. Therefore, the drying of kale ensures its preservation, easy storage, transport and availability over the year. Drying is one of the oldest methods of preserving plant materials. This method, due to the fact that removes almost all water from the product, allows the microbiological safety of the product to be ensured (Ghaboos et al. 2016; Saengrayap et al. 2015).

Dried foods are low water activity ranging from 0.03 to 0.7 (Chitrakar et al. 2019). A decreased water activity inhibits the growth of most bacteria, yeasts, and molds, which are unable to grow below 0.87, 0.88, and 0.80, respectively (Beuchat et al. 2013). Additionally, the blanching of vegetables before drying destroys the non-sporulating pathogens such as *E. coli* and *Salmonella* (International Commission on Microbiological Specifications for Foods 2005).

It should be emphasized that dried plant products are produced in many countries around the world, but there are increasing warnings about the presence of pathogens such as *Salmonella.* There are reported cases where many food borne illnesses were caused by the consumption of dried foods contaminated with *Salmonella* spp., *Cronobacter* spp., *Staphylococcus* spp. and *E. coli*. The bacterial population commonly isolated from dried vegetables include lactic acid bacteria, *Enteroccoccus faecalis*, *Staphylococci*, spores of *Bacillus* spp., yeasts and molds (*Penicillium* and *Aspergillus* spp.). With regard to pathogens in dried vegetables, the vegetative cells are rarely prezent (Chitrakar et al. 2019). Food safety affects food production and processing. Therefore, it is important the implementation of food safety programs in the food industry and improvements in hygiene education for people working with foods (El Sheikha 2016). The importance of the quality of the raw material for drying, production hygiene and appropriate packaging should be highlighted. The packaging of dehydrated vegetables must protect the product against moisture, air, dust, microorganisms, foreign odour, insects and rodents throughout storage. Heat and light also have a negative effect on the quality of the dried food (Sharangi and Datta 2015).

Convection drying by hot air (AD) is the most popular and traditional method of drying. However, while in contact with oxygen present in the air, the product is exposed to high temperatures for a long time. This, in turn, reduces the content of some valuable components, easily oxidised under such conditions (Ghaboos et al. 2016; Shende and Datta 2019). However, the application of lower temperatures, for example, during freeze-drying (FD), makes it is possible to produce high-quality dried products (Cheaib et al. 2018). Freeze drying, one of the more advanced drying methods, is a method which removes water from the frozen material mainly by sublimation. In this method, shrinkage is eliminated, and minimum losses of flavour, aroma and vitamins, along with near-perfect preservation results, are obtained (Antal et al. 2014). Compared to conventional drying, the key benefits of freeze-drying include the following: the retention of morphological, biochemical and immunological properties, high yield, long shelf life and reduced weight for storage and shipping (Ciurzyńska and Lenart 2011).

Dried products are being increasingly used in industries related to, for example, confectionery, bakery products and the production of functional foods. Drying technologies and the storage conditions of dried products should be adjusted to maintain the quality of dehydrated products. However, there is little in the literature on the effects of the pre-treatment of vegetables before drying and the storage time and temperature of dehydrated vegetables. Therefore, the aim of this study was to determine the effects of pre-treatment (blanching), the method of drying (air or freeze-drying), and 12 month storage at two temperatures on the content of ash, minerals (P, K, Ca, Mg, Na, Fe, Zn, Mn, Cu, Cr, Ni, Pb and Cd), vitamins (B_1_ and B_2_) and tocopherols (α-, ß- and γ-tocopherol) in kale leaves (*Brassica oleracea* L var. *acephala*).

## Materials and methods

### Materials

The study material consisted of raw kale (*Brassica oleracea* L. var**.** *acephala*) leaves; leaves after blanching; leaves after air-drying (AD); and leaves after freeze-drying (FD). The dried products were evaluated directly after processing (0 months) and after 12 months of storage at ambient temperature, 18–20 °C (A) and cold temperature, 8–10 °C (C).

The kale cultivar under investigation was Winterbor F_1_, produced by the Dutch firm Bejo Zaden b.v. (Netherlands). This cultivar is classed among those most resistant to disease, yellowing of lower leaves and frost damage. Its leaves are dark green and strongly corrugated. The kale was grown from seedlings planted in an experimental field located in southern Poland. Seedlings were planted at spacings of 0.5 × 0.5 m in late June. Mineral fertilization was applied according to the fertility of the soil and the nutritional requirements of the crop.

Kale was obtained from a single harvest during the last five days of September, 14 weeks after the seedlings were planted. The harvest consisted of cutting whole plants and removing unusable leaves. Leaves suitable for technological processing, i.e., not discoloured or damaged by pests or disease, then had their main veins removed. A random sample of approximately 5 kg was taken for analysis of chemical composition of the raw material. Part of the remaining material was then blanched before the whole was dried.

### Drying technology

Non-blanched (NB) and blanched (B) raw leaves of kale were dried to the predetermined humidity level (below 5%) by air or freeze-drying. The process of drying was preceded by the following preliminary treatments: removing the main vein, washing, cutting into 2 cm long pieces (Korus 2011).

Kale was blanched in a stainless steel vessel for 2.5 min at 96–98 °C, the proportion of water to the raw material being 5:1 by weight. After blanching, the material was cooled in cold water and left to drain on sieves for 30 min to remove excess water.

Air drying (AD) was carried out using Food dehydrator Profi Line (Hendi UK Ltd.), Model No: 229026 (1000 W). The weight of leaves on 1 m^2^ of sieves was 4.0 kg. Cut kale leaves were dried at a temperature of 55 °C, 5.0 h.

Freeze-drying (FD) was preceded by freezing cut kale leaves for 80 min at − 40 °C. Drying was carried out in a CHRIST Gamma 1–16 LCS lyophilizing cabinet. The weight of leaves on 1 m^2^ of trays was 4.0 kg. The process of freeze-drying was begun at an initial temperature of − 25 °C. The process was terminated after 30 h of drying, when the temperature of the dried material and that of the trays in the lyophilizing chamber reached 30 °C.

After drying, the material was mixed and packed in air-tight containers (glass twist-off jars 2.0 L in volume). The dried material was stored at two different temperatures until analysis:

- ambient temperature, 18–20 °C (A),
- cold temperature, 8–10 °C (C).

### Chemical determination

The ash content was determined by incinerating at 460 °C in an L 9/S 27 furnace oven (Nabertherm GmbH, Lilienthal, Germany) (Horwitz 2006). For the determination of individual mineral elements, the mineralisation of the material was carried out in a 3:1 mixture of nitric and perchloric acids. A 50 g portion of the material and 30 mL of the acid mixture were placed in 250 mL test tubes belonging to a Tecator Kjeltec Auto Plus II mineralisation set (FOSS, Hillerød, Denmark). The treated samples were left until the next day when complete mineralisation had occurred. The mineralised samples were diluted with ultra-pure water to a volume of 100 mL and filtered into a dry flask. The content of the individual elements in the solutions was determined using a Jobin Yvon JY 238 Ultrace inductively coupled argon plasma emission spectrometer (Horiba, Kyoto, Japan). The level of P was determined by the method given in AOAC (Horwitz 2006).

The vitamin B_1_ and B_2_ content was determined according to HPLC methods (EN 14122:2014 (2014); EN 14152:2014 (2014)). Thiamine and riboflavin were detected simultaneously using a Merck liquid chromatograph with a fluorescence detector. Analysis was carried out on an Onyx Monolithic C18 column (100 × 4.6 mm) with a pre-column. The fluorescence measurement was made at excitation and emission wavelengths of 360 nm and 503 nm, respectively. Water (w) and acetonitrile (ac) (*t* = 0 w/ac 88/12; *t* = 12 w/ac 0/100) were used as the mobile phase with gradient elution at a flow rate of 1 mL/min.

The tocopherol content was determined according to a modification of the method of Katsanidis and Addis (1999) using normal phase HPLC. Tocopherols were extracted with hexane mixed with BHT. The analysis was carried out on a Merck liquid chromatograph with a fluorescence detector. The sample was injected onto a LUNA NH_2_ column (250 × 4.6 mm) with a pre-column. Isocratic elution was carried out using a mixture of n-hexane and 2-propanol (95:5) at a flow rate of 2.5 mL/min. The wavelengths of excitation and emission were 290 nm and 330 nm, respectively.

### Statistical analysis

The obtained data were analyzed by one-factor analysis of variance, with three experimental replications. The Snedecor F and Student’s t tests, as well as the least significant difference (LSD), were calculated at probability levels of *p* < 0.05. Statistica 12.0 (StatSoft Inc., Tulsa, USA) software was used.

## Results and discussion

### Effect of blanching on the level of minerals and vitamins in kale leaves

Dark green vegetables, including kale, are a good source of minerals (Dias 2019). Kale added to a cereal-based diet enhances the intake of essential minerals to combat micronutrient malnutrition (Migliozzi et al. 2015). The present study revealed that the average ash content in fresh kale leaves was 10.1 g per 100 g of dry matter (Table 1). The main ash components were potassium (2613 mg/100 g), calcium (1346 mg/100 g), phosphorus (432 mg/100 g) and magnesium (129 mg/100 g). The remaining minerals were present in quantities below 100 mg/100 g dry weight (Table 1). It should, however, be noted that not all minerals are considered essential for the body to function as normal. Heavy metals pose a health risk due to their ability to accumulate in the body and long biological half-life. For example lead (Pb), cadmium (Cd) and arsenic (As) have a direct effect on the kidneys, and they are particularly nephrotoxic, even at ‘normal’ levels (Sabath and Robles-Osorio 2012). Fresh kale leaves contained 0.061 mg of lead and 0.097 mg of cadmium per 100 g dry matter (Table 1).

**Table 1:** Content of ash (g/100 g), macro- and microelements (mg/100 g) in raw and blanched leaves of kale

Item	Raw	Blanched	LSD *p* < 0.05
Ash	10.1 ± 0.18	7.9 ± 0.12	2.15
Potassium	2613 ± 161	1612 ± 122	411.9
Calcium	1346 ± 43	1301 ± 25	ns
Phosphorus	432 ± 13	358 ± 17	73.2
Magnesium	129 ± 9	112 ± 11	16.2
Sodium	51.6 ± 5	44.5 ± 7	6.22
Iron	15.0 ± 2.2	13.2 ± 1.9	1.95
Zinc	9.01 ± 1.81	8.57 ± 1.71	ns
Manganese	6.46 ± 1.11	6.07 ± 1.21	ns
Copper	7.13 ± 1.62	5.45 ± 1.03	1.58
Chrome	0.164 ± 0.015	0.156 ± 0.011	0.0072
Nickel	0.177 ± 0.010	0.164 ± 0.009	0.0124
Lead	0.061 ± 0.005	0.055 ± 0.004	0.0052
Cadmium	0.097 ± 0.007	0.086 ± 0.006	0.0109

Values are presented as mean value ± SD (*n* = 3), in dry matter

*ns* not significant

The high vitamin content in kale is the factor that distinguishes this vegetable from other *Brassica* vegetables. The examined kale had 0.828 mg vitamin B_1_ and 1.533 mg vitamin B_2_ in 100 g of dry matter. In turn, the total tocopherol content was 16.55 mg per 100 g dry matter, including α-tocopherol (80%), γ-tocopherol (17%) and β-tocopherol (3%) (Table 2).

**Table 2:** Content of selected from B-group vitamins and tocopherols (mg/100 g) in raw, and blanched leaves of kale

Item	Raw	Blanched	LSD *p* < 0.05
Vitamin B_1_	0.828 ± 0.014	0.767 ± 0.005	0.0227
Vitamin B_2_	1.533 ± 0.013	0.997 ± 0.020	0.0382
α-tocopherol	13.30 ± 0.16	12.20 ± 0.15	0.831
ß-tocopherol	0.506 ± 0.070	0.402 ± 0.080	0.0159
γ-tocopherol	2.741 ± 0.012	1.519 ± 0.009	0.0241
Total tocopherols	16.55 ± 0.22	14.12 ± 0.31	0.742

Values are presented as mean value ± SD (*n* = 3), in dry matter

Thermal blanching is often a necessary operation preceding the main vegetable preservation process. The blanching treatment, by inactivating enzymes, including polyphenol oxidase (PPO) and peroxidase (POD), and removing air, has a beneficial effect on the quality of dried products (Garba et al. 2015; Xiao et al. 2017). In addition, blanching increases the drying rate by loosening vegetable tissue (Deng et al. 2019). This operation influences the loss of water-soluble compounds, for example, minerals and heat-sensitive vitamins. Santos et al. (2003) found that the decrease in the mineral content of vegetable leaves depends on the species and the time of the thermal treatment in water. The present study found that the blanching of kale leaves reduced the levels of ash on average by 21% and minerals by 3–38%, with insignificant differences for calcium, zinc and magnesium (Table 1).

In turn, the losses of thiamine and riboflavin were, respectively, 9% and as much as 36%. Lisiewska et al. (2003) recorded significant losses of both thiamine (45%) and riboflavin (33%) in the fresh weight of blanched fennel leaves; similar losses were observed by Słupski (2012) in common beans. The main reasons for the reduction observed in the contents of the examined vitamins are their water solubility and thermal destruction (Rickman et al. 2007). As for tocopherols, the largest losses due to blanching kale leaves were observed for γ-tocopherol (45%) and β-tocopherol (21%). Losses of α-tocopherol were smaller, not exceeding 10% (Table 2). The nature of factors influencing the extent of changes resulting from heat treatment in water is complex. These factors include the ratio of vegetable weight to water during blanching and the contact surface of the raw material and medium (Lešková et al. 2006).

### Effect of drying on the level of minerals and vitamins in kale leaves

Recently, there has been a growing consumer demand for convenient and ready-to-eat food. Dried fruits and vegetables are components of many convenience products, whose production and supply are currently increasing. Dried kale can be used, for example, for the production of powders considered as a good source of nutrients; it can also be a component of dietary supplements (Salehi and Aghajanzadeh 2020). Directly after air-drying, the products obtained had 9.84–9.86 g of ash, while the ash content in freeze-dried products was 7.80–7.90 g per 100 g dry matter (Table 3). In the dried products from blanched leaves, the level of ash was lower, on average by 21%, compared to those obtained from unblanched leaves, regardless of the drying method. After one year's storage, the ash level remained practically unchanged.

**Table 3:** Content of ash (g/100 g) and macroelements (mg/100 g) in dried leaves of kale

Type of dried product	Ash	Potassium	Calcium	Phosphorus	Magnesium	Sodium
*0 months stored:*						
AD, NB	9.84 ± 0.39	1946 ± 201	1138 ± 82	399 ± 9	112 ± 9	41.3 ± 1.1
FD, NB	9.86 ± 0.33	2203 ± 123	1149 ± 106	402 ± 16	121 ± 8	48.1 ± 1.8
AD, B	7.80 ± 0.20	1575 ± 102	1220 ± 68	352 ± 19	109 ± 8	40.5 ± 0.9
FD, B	7.90 ± 0.04	1595 ± 90	1256 ± 122	356 ± 17	118 ± 3	43.6 ± 1.6
LSD *p* < 0.05	0.423	202.3	ns	38.4	13.9	3.87
*After 12 months stored:*						
AD, NBA	9.65 ± 0.31	1899 ± 92	1052 ± 51	368 ± 11	104 ± 5	36.9 ± 2.0
AD, NBC	9.76 ± 0.33	1907 ± 87	1108 ± 90	382 ± 15	111 ± 6	37.9 ± 1.9
FD, NBA	9.77 ± 0.38	2045 ± 71	1088 ± 17	393 ± 12	110 ± 8	46.7 ± 2.4
FD, NBC	9.79 ± 0.27	2128 ± 99	1121 ± 98	397 ± 17	115 ± 8	47.0 ± 2.2
LSD *p* < 0.05	ns	135.5	ns	ns	ns	3.29
AD, BA	7.76 ± 0.36	1512 ± 23	1200 ± 96	348 ± 15	102 ± 4	38.2 ± 2.7
AD, BC	7.76 ± 0.17	1524 ± 48	1215 ± 73	350 ± 7	103 ± 6	38.7 ± 1.2
FD, BA	7.79 ± 0.32	1585 ± 93	1205 ± 85	353 ± 22	103 ± 3	42.0 ± 1.1
FD, BC	7.79 ± 0.42	1590 ± 90	1225 ± 84	355 ± 20	105 ± 5	42.9 ± 1.0
LSD *p* < 0.05	ns	65.6	ns	ns	ns	2.79
Total LSD *p* < 0.05	0.430	151.2	ns	32.7	9.6	3.21

Values are presented as mean value ± SD (*n* = 3), in dry matter

Type of product: *AD, NB* air-dried from non-blanched kale; *FD, NB* freeze-dried from non-blanched kale; *AD, B* air-dried from blanched kale, *FD, B* freeze-dried from blanched kale, *AD, NBA* air-dried from non-blanched kale, stored in ambient temperature, *AD, NBC* air-dried from non-blanched kale, stored in cold temperature, *FD, NBA* freeze-dried from non-blanched kale, stored in ambient temperature, *FD, NBC* freeze-dried from non-blanched kale, stored in cold temperature, *AD, BA* air-dried from blanched kale, stored in ambient temperature, *AD, BC* air-dried from blanched kale, stored in cold temperature, *FD, BA* freeze-dried from blanched kale, stored in ambient temperature, *FD, BC* freeze-dried from blanched kale, stored in cold temperature

*ns* not significant

In the dried products, potassium (1575–2203 mg/100 g) and calcium (1138–1256 mg/100 g) were the minerals with the highest content (Table 3). The amounts of phosphorus (352–402 mg) and magnesium (108–121 mg) were also significant. Moreover, these products had 40.5–48.1 mg sodium, 13.0–14.9 mg iron, 8.52–10.51 mg zinc, 5.85–6.02 mg manganese and 5.15–6.91 mg copper in 100 g (Tables 3, 4). Dried products from blanched leaves, compared to those obtained from unblanched ones, had lower levels of ash (20–21%), macroelements (2–29%) and microelements (2–25%), since after blanching, the leaves had less of the aforementioned components. This is congruent with the opinion of other authors about losses of water-soluble components due to blanching (Huang et al. 2016). Throughout storage, the level of macro- and micronutrients in the dried products did not change significantly. These components are regarded as fairly stable during storage, which agrees with our findings. It was also found that storage temperature had no significant effect on the level of macro- and microelements.

**Table 4:** Content of microelements (mg/100 g) in dried leaves of kale

Type of dried product	Iron	Zinc	Manganese	Copper	Chrome	Nickel	Lead	Cadmium
*0 months stored:*								
AD, NB	14.9 ± 0.82	8.79 ± 0.48	5.26 ± 0.41	6.91 ± 0.14	0.160 ± 0.006	0.174 ± 0.006	0.059 ± 0.004	0.097 ± 0.003
FD, NB	14.5 ± 0.52	8.89 ± 0.28	5.99 ± 0.34	6.69 ± 0.26	0.158 ± 0.010	0.161 ± 0.018	0.060 ± 0.003	0.086 ± 0.005
AD, B	13.0 ± 0.15	8.49 ± 0.29	5.02 ± 0.08	5.16 ± 0.27	0.152 ± 0.010	0.160 ± 0.019	0.053 ± 0.002	0.085 ± 0.002
FD, B	13.1 ± 2.60	8.52 ± 0.43	5.85 ± 0.21	5.15 ± 0.19	0.146 ± 0.022	0.156 ± 0.010	0.055 ± 0.003	0.083 ± 0.003
LSD *p* < 0.05	0.8	0.585	0.444	0.341	ns	0.0221	0.0062	0.0055
*After 12 months stored:*								
AD, NBA	14.5 ± 0.93	7.93 ± 0.68	4.58 ± 0.33	6.34 ± 0.42	0.141 ± 0.008	0.173 ± 0.007	0.046 ± 0.003	0.096 ± 0.002
AD, NBC	14.5 ± 0.90	7.72 ± 0.34	4.72 ± 0.28	6.57 ± 0.38	0.135 ± 0.010	0.172 ± 0.006	0.044 ± 0.003	0.093 ± 0.005
FD, NBA	14.1 ± 0.29	7.88 ± 0.16	5.86 ± 0.30	6.60 ± 0.23	0.130 ± 0.008	0.161 ± 0.020	0.059 ± 0.002	0.087 ± 0.005
FD, NBC	14.2 ± 0.38	7.94 ± 0.20	5.86 ± 0.33	6.61 ± 0.25	0.131 ± 0.011	0.160 ± 0.018	0.059 ± 0.004	0.086 ± 0.002
LSD *p* < 0.05	ns	ns	0.475	ns	0.0145	0.0124	0.0035	ns
AD, BA	12.5 ± 0.93	8.23 ± 0.37	5.00 ± 0.08	5.01 ± 0.31	0.155 ± 0.007	0.156 ± 0.017	0.060 ± 0.002	0.084 ± 0.004
AD, BC	12.7 ± 0.90	8.26 ± 0.37	4.98 ± 0.16	5.06 ± 0.21	0.153 ± 0.009	0.159 ± 0.008	0.059 ± 0.004	0.085 ± 0.002
FD, BA	12.8 ± 0.29	8.36 ± 0.19	5.37 ± 0.39	5.05 ± 0.22	0.150 ± 0.010	0.154 ± 0.009	0.055 ± 0.002	0.083 ± 0.003
FD, BC	12.9 ± 0.38	8.34 ± 0.33	5.43 ± 0.44	5.10 ± 0.26	0.147 ± 0.007	0.155 ± 0.010	0.054 ± 0.003	0.082 ± 0.004
LSD *p* < 0.05	ns	ns	ns	ns	0.0129	ns	ns	ns
Total LSD *p* < 0.05	0.83	0.523	0.424	0.371	0.0161	0.0193	0.0047	0.0034

Values are presented as mean value ± SD (*n* = 3), in dry matter

Abbreviations of type of dried product are the same as explained in footnote of Table 3

*ns*- not significant

Of the micronutrients examined, zinc was found in the largest amount (8.52–8.89 mg/100 g), while with regard to the metals considered particularly dangerous to human health, it was nickel (0.156–0.1774 mg/100 g) and chromium (0.146–0.160 mg/100 g). Lead and cadmium were below 0.1 mg/100 g dry matter (Table 4). It should be highlighted that air-dried products, but only from unblanched raw material, had higher levels of lead and cadmium, compared to freeze-dried products, while the chromium and nickel contents were comparable in both products.

In contrast to minerals, the majority of the vitamins occurring in vegetables are sensitive to light, oxygen and especially to elevated temperatures (Santos et al. 2012). Drying lowered the vitamin content in kale leaves, and in this case the extent of the losses depended on the drying method. In freeze-dried products, the level of all the analysed vitamins was significantly higher (Table 5). During drying, vitamin losses were higher in the dried products obtained from the unblanched kale leaves, being as follows: 3–13% for vitamin B_1_, 30–49% for vitamin B_2_ and 15–36% for total tocopherols. Immediately after drying, however, the products from the blanched raw material contained, in general, slightly less vitamins B_1_ and B_2_, compared to those from unblanched material, while their level of tocopherols was higher. It should be emphasised that after one year of storage, vitamins were better retained in the products obtained from blanched leaves. This result is consistent with the findings of Ramesh et al. (2001), who found that blanching led to lower vitamin losses in the samples blanched prior to drying than in unblanched leaves. In addition, vitamin losses were smaller in the dried kale kept at refrigeration temperature.

**Table 5:** Content of selected from B-group vitamins and tocopherols (mg/100 g) in dried leaves of kale

Type of dried product	Vitamin B_1_	Vitamin B_2_	α-tocopherol	ß-tocopherol	γ-tocopherol	Total tocopherols
*0 months stored:*						
AD, NB	0.722 ± 0.029	0.776 ± 0.049	10.05 ± 0.41	0.12 ± 0.01	0.42 ± 0.03	10.59 ± 0.39
FD, NB	0.803 ± 0.035	1.073 ± 0.059	11.41 ± 0.34	0.35 ± 0.02	2.35 ± 0.15	14.11 ± 0.48
AD, B	0.685 ± 0.018	0.734 ± 0.032	11.45 ± 0.43	0.20 ± 0.02	0.82 ± 0.07	12.47 ± 0.50
FD, B	0.757 ± 0.050	0.962 ± 0.024	12.07 ± 0.25	0.36 ± 0.04	1.30 ± 0.09	13.73 ± 0.23
LSD *p* < 0.05	0.0542	0.0667	0.562	0.035	0.145	0.641
*After 12 months stored:*						
AD, NBA	0.602 ± 0.038	0.685 ± 0.034	8.44 ± 0.33	0.04 ± 0.01	0.32 ± 0.03	8.78 ± 0.36
AD, NBC	0.632 ± 0.038	0.712 ± 0.031	8.87 ± 0.29	0.06 ± 0.01	0.38 ± 0.02	9.31 ± 0.30
FD, NBA	0.694 ± 0.036	0.902 ± 0.030	10.86 ± 0.42	0.28 ± 0.02	2.14 ± 0.12	13.28 ± 0.53
FD, NBC	0.723 ± 0.037	0.944 ± 0.033	10.99 ± 0.42	0.30 ± 0.02	2.24 ± 0.20	13.53 ± 0.54
LSD *p* < 0.05	0.0571	0.0490	0.567	0.021	0.180	0.682
AD, BA	0.624 ± 0.038	0.705 ± 0.027	9.86 ± 0.32	0.10 ± 0.02	0.56 ± 0.03	10.52 ± 0.34
AD, BC	0.653 ± 0.037	0.733 ± 0.030	10.31 ± 0.42	0.17 ± 0.02	0.72 ± 0.03	11.20 ± 0.44
FD, BA	0.699 ± 0.032	0.944 ± 0.025	11.74 ± 0.57	0.20 ± 0.02	1.07 ± 0.06	13.01 ± 0.53
FD, BC	0.746 ± 0.038	0.950 ± 0.022	12.07 ± 0.25	0.31 ± 0.02	1.19 ± 0.11	13.57 ± 0.31
LSD *p* < 0.05	0.0560	0.0400	0.745	0.037	0.100	0.637
Total LSD *p* < 0.05	0.0511	0.0524	0.537	0.030	0.134	0.599

Values are presented as mean value ± SD (*n* = 3), in dry matter

Abbreviations of type of dried product are the same as explained in footnote of Table 3

The degree of degradation will vary according to the vitamin and can also be affected by the processing and storage time to which the vegetable is submitted. Monitoring the vitamin content during processing and storage is of great importance to food technologists and consumers to assure the nutritious value of foods, and also for quality assurance purposes and regulatory compliance (Santos et al. 2012). In the process of drying, a high temperature is an important and characteristic feature, but it reduces the quality of the final product. Lower temperatures used, for example, in freeze-drying have a great potential for giving an improvement in the quality of dried products (Cheaib et al. 2018). Marić et al. (2020) noted that lyophilisation was less destructive to the original properties of the analysed root vegetables than convective drying, while Beaudry et al. (2004) pointed out the better quality of freeze-dried products; this technique, however, involves higher processing costs.

## Conclusion

Drying plant materials with high water content extends their durability by reducing water activity and limiting enzymatic and microbiological processes. Dried products from kale can be components of other products, since they contain high amounts of minerals, B-group vitamins and tocopherols. When applied as food ingredients, they can give food new organoleptic features and increase its nutritional value. Each type of raw material, however, requires the use of specific conditions, the determination of which significantly affects the final quality of the obtained dried material. The application of improper drying parameters, especially temperature, can induce a number of undesirable changes, including losses in nutrients. However, particular attention should be given to the quality of the raw material, hygiene of production and storage conditions.

Our studies revealed that a high quality of dried products, especially vitamin retention, is determined by the pre-treatment (blanching), the drying method and the storage conditions. As such products have a relatively long shelf-life, blanching the raw material as a pre-treatment step produces a beneficial effect. Although the blanching of kale leaves led to losses in minerals and vitamins at this initial stage of processing, the losses observed in the products obtained from blanched leaves throughout their storage were smaller, especially with regard to vitamins B_1_ and B_2_, and the tocopherols, compared to the samples from unblanched leaves. In addition, lower losses of vitamins were observed in dried kale stored at a lower temperature.

Generally, dried products from kale can be components of other products, since they contain high amounts of minerals, B-group vitamins and tocopherols. When applied as food ingredients, they can give food new organoleptic features and increase its nutritional value.

With regard to dried vegetables, a food safety control policy and stringent quality control measures should be developed. In order to ensure the microbiological safety of dried food, it is important to take into account the quality of raw materials, processing and storage conditions.
